# Gut microbiome in endometriosis: a cohort study on 1000 individuals

**DOI:** 10.1186/s12916-024-03503-y

**Published:** 2024-07-18

**Authors:** Inmaculada Pérez-Prieto, Eva Vargas, Eduardo Salas-Espejo, Kreete Lüll, Analuce Canha-Gouveia, Laura Antequera Pérez, Juan Fontes, Andres Salumets, Reidar Andreson, Oliver Aasmets, Estonian Biobank research team, Metspalu Mait, Metspalu Andres, Milani Lili, Esko Tõnu, Katrine Whiteson, Elin Org, Signe Altmäe

**Affiliations:** 1 Department of Biochemistry and Molecular Biology I, Faculty of Sciences, University of Granada, Granada, Spain; 2 Instituto de Investigación Biosanitaria Ibs.GRANADA, Granada, Spain; 3 Systems Biology Unit, Department of Experimental Biology, Faculty of Experimental Sciences, University of Jaen, Jaen, Spain; 4 Institute of Genomics, Estonian Genome Centre, University of Tartu, Tartu, Estonia; 5 Department of Physiology, Faculty of Veterinary, University of Murcia, IMIB-Arrixaca, Campus Mare Nostrum, Murcia, Spain; 6 Department of Computer Engineering, Automation and Robotics, University of Granada, Granada, Spain; 7 U. Reproducción, UGC Laboratorio Clínico y UGC Obstetricia y Ginecología. HU Virgen de Las Nieves, Granada, Spain; 8 Competence Centre On Health Technologies, Tartu, Estonia; 9 Division of Obstetrics and Gynecology, Department of Clinical Science, Intervention and Technology, Karolinska Institutet and Karolinska University Hospital, Stockholm, Sweden; 10 Department of Obstetrics and Gynaecology, Institute of Clinical Medicine, University of Tartu, Tartu, Estonia; 11 Institute of Molecular and Cell Biology, University of Tartu, Tartu, Estonia; 12 Institute of Genomics, Estonian Genome Center, University of Tartu, Tartu, Estonia; 13 School of Biological Sciences, University of California, Irvine, CA USA

**Keywords:** Endometriosis, Estrobolome, Gut microbiota, Metagenomics, Microbiome, Microbiota, Shotgun sequencing

## Abstract

**Background:**

Endometriosis, defined as the presence of endometrial-like tissue outside of the uterus, is one of the most prevalent gynecological disorders. Although different theories have been proposed, its pathogenesis is not clear. Novel studies indicate that the gut microbiome may be involved in the etiology of endometriosis; nevertheless, the connection between microbes, their dysbiosis, and the development of endometriosis is understudied. This case–control study analyzed the gut microbiome in women with and without endometriosis to identify microbial targets involved in the disease.

**Methods:**

A subsample of 1000 women from the Estonian Microbiome cohort, including 136 women with endometriosis and 864 control women, was analyzed. Microbial composition was determined by shotgun metagenomics and microbial functional pathways were annotated using the Kyoto Encyclopedia of Genes and Genomes (KEGG) database. Partitioning Around Medoids (PAM) algorithm was performed to cluster the microbial profile of the Estonian population. The alpha- and beta-diversity and differential abundance analyses were performed to assess the gut microbiome (species and KEGG orthologies (KO)) in both groups. Metagenomic reads were mapped to estrobolome-related enzymes’ sequences to study potential microbiome-estrogen metabolism axis alterations in endometriosis.

**Results:**

Diversity analyses did not detect significant differences between women with and without endometriosis (alpha-diversity: all *p*-values > 0.05; beta-diversity: PERMANOVA, both *R* ^2^ < 0.0007, *p*-values > 0.05). No differential species or pathways were detected after multiple testing adjustment (all FDR *p*-values > 0.05). Sensitivity analysis excluding women at menopause (> 50 years) confirmed our results. Estrobolome-associated enzymes’ sequence reads were not significantly different between groups (all FDR *p*-values > 0.05).

**Conclusions:**

Our findings do not provide enough evidence to support the existence of a gut microbiome-dependent mechanism directly implicated in the pathogenesis of endometriosis. To the best of our knowledge, this is the largest metagenome study on endometriosis conducted to date.

**Supplementary Information:**

The online version contains supplementary material available at 10.1186/s12916-024-03503-y.

## Background

Endometriosis, defined as the growth of endometrial-like tissue outside of the uterine cavity, is a common gynecologic disease, affecting approximately 5–10% of reproductive-aged women [1]. Endometrial lesions cause a chronic inflammatory condition associated with a wide range of reported symptoms, including dysmenorrhea, pelvic pain, dyspareunia, and infertility [2, 3]. Because these symptoms are associated with other conditions, diagnosing endometriosis requires laparoscopic examination with excisional biopsy for definitive pathology confirmation, which leads to a long diagnostic delay or frequent misdiagnosis. Although endometriosis is a widespread and burdening reproductive disorder, it has been historically understudied. Notably, proposed hypotheses such as retrograde menstruation, coelomic metaplasia, and Müllerian remnants do not explain the etiology of all the different phenotypes of endometriosis (i.e., superficial, ovarian and deep infiltrating endometriosis) [4]. Thus, endometriosis represents an important public health concern with substantial effects on the quality of life of millions of women globally [5, 6].

The microbiome refers to the collection of genomes of the microorganisms (bacteria, viruses, fungi, protozoa, and archaea) that inhabit a particular environment [7]. Particularly, the human gastrointestinal system is the most diverse microbiome within the human body, being colonized by trillions of microbes that play key roles in regulating host physiological functions [8, 9]. Indeed, a healthy balanced gut microbiome is crucial for nutrient absorption, gut epithelial barrier integrity, immune system work, and other body functions [10, 11]. Nevertheless, compositional and functional perturbations in the microbiome could lead to an unstable state called dysbiosis, which is linked to different chronic conditions such as obesity, type-2 diabetes, cancer, inflammatory bowel diseases, and neurological and reproductive diseases, among others [12–16].

Extensive research associates the gut microbiome with circulating levels of estrogens through the secretion of β-glucuronidase, an enzyme that deconjugates estrogen into its active metabolized form [17]. The estrobolome term encapsulates the gut gene repertoire of microbial origin capable of metabolizing estrogens leading to the stimulation of epithelial proliferation throughout the female reproductive tract. Therefore, estrogen dysregulation has been shown to drive proliferative diseases such as endometriosis along with its main comorbidities like infertility and pelvic pain [18]. Indeed, the use of estrogen-progestins and progestins is the first-line medical treatment of endometriosis due to their safety, tolerability, and favorable cost profile, although they are often ineffective and may lead to unwanted side effects [19]. Hence, to date, there is no cure for endometriosis and new non-hormonal therapeutic approaches are becoming increasingly necessary [20].

Considering the influence of the gut microbiome on immunomodulation and estrogen metabolism, alongside the estrogen-driven inflammatory state in endometriosis, a potential role of the gut microbiome in the pathogenesis of the disease has been proposed [18, 21]. Recent studies suggest that gut dysbiosis induces an increment in the estrogen circulating levels, which may contribute to the hyper-estrogenic environment that promotes the progression of endometriosis [22]. Nevertheless, the connection between microbes, their dysbiosis, and the development of endometriosis remains unexplored. Research on the gut microbiome in endometriosis would enable the identification of novel biomarkers for noninvasive diagnostic and therapeutic approaches to identify and treat women with endometriosis earlier [23].

This study aimed to analyze and compare the gut microbiome profiles in a large cohort of women with and without endometriosis, to identify microbial signatures and pathways potentially associated with the development of the disease. We also explored the link between the estrogen metabolism and endometriosis by analyzing microbial enzyme reads of the estrobolome between women with endometriosis and controls.

## Methods

### Study population

This case–control study included 1000 women of the Estonian Microbiome (EstMB) cohort (age = 45.61 ± 10.36 years; BMI = 25.67 ± 5.59), a volunteer-based sub-cohort of the Estonian Biobank (EstBB) created in 2017 with the objective of enriching the previous existing data with microbiome data [24, 25]. Out of the 1000 women included in this study, two groups were established: the endometriosis group comprised of 136 patients diagnosed with this disease, and the remaining 864 individuals were grouped into the control group. Since endometriosis has been reported to have a high degree of comorbidity with other disorders [26–28], control women were not diagnosed with any of the most prevalent comorbidities of endometriosis (systemic lupus erythematosus, rheumatoid arthritis, autoimmune thyroiditis, celiac disease, multiple sclerosis, and irritable bowel syndrome). Endometriosis was confirmed by diagnostic laparoscopy, and the cases were identified from the electronic health record data based on the ICD-10 code (N80). Self-reported data on diseases, medications, medical procedures, health-related behaviors in lifestyle, diet, physical activity, living environment, delivery mode, and stool characteristics (Bristol stool scale) were collected for each participant [25].

### Sample collection and DNA extraction

The sample collection took place between 2017 and 2019. Fresh stool samples were collected by the participants immediately after defecation with a sterile Pasteur pipette, placing the samples inside a polypropylene conical 15-ml tube and stored in the fridge (+ 4 °C) until transportation. The sample was subsequently delivered to the study center where it was stored at − 80 °C until processing.

For genomic DNA isolation, microbial DNA was extracted using the QIAamp DNA Stool Mini Kit (Qiagen, Germany). Approximately 200 mg of stool was used as starting material for DNA extraction following the manufacturer’s instructions. Next, the extracted DNA was quantified using the Qubit 2.0 Fluorometer with dsDNA Assay Kit (Thermo Fisher Scientific). Sequencing libraries were generated using NEBNext® Ultra™ DNA Library Prep Kit for Illumina (NEB, USA) following the manufacturer’s recommendations. Briefly, 1 μg DNA per sample was used as input material, and index codes were added to attribute sequences to each sample. Each DNA sample was fragmented by sonication to an average size of 350 bp, DNA fragments were end-polished, A-tailed, and ligated with the full-length adaptor for Illumina sequencing with further PCR amplification. Finally, PCR products were purified (AMPure XP system) and libraries were analyzed for size distribution by Agilent2100.

### Metagenomics analyses

The shotgun metagenomic paired-end sequencing was performed by Novogene Bioinformatics Technology Co., Ltd. in the Illumina NovaSeq6000 platform resulting in 4.62 ± 0.44 Gb of data per sample (insert size, 350 bp; read length, 2 × 250 bp). Metagenomic analysis was performed as previously described [25]. Briefly, the reads were trimmed for quality and adapter sequences. The host reads that aligned to the human genome were removed with SOAP2.21 (parameters: -s 135 -l 30 -v 7 -m 200—× 400) [29]. Quality-controlled data of each sample was then used for metagenomic assembly using SOAPdenovo (v. 2.04, parameters: -d 1 -M 3 -R -u –F) [30]. Next, SOAP2.21 was used to map the clean data of each sample to the assembled scaftigs (i.e., continuous sequences within scaffolds). Unutilized paired-end reads of each sample were compiled together for mixed assembly. MetaGeneMark (v.3.38) was used to carry out gene prediction (gene length > 100 bp) based on the scaftigs (≥ 500 bp), which were assembled by single and mixed samples. CD-HIT (v.4.6) was used to dereplicate the predicted genes based on 95% identity and 90% coverage to generate the gene catalogs (parameters: -c 0.95, -G 0, -aS 0.9, -g 1, -d 0) [31]. The longest dereplicated gene was defined as the representative gene (i.e., unigene). SoapAligner [32] (v.2.21, parameters: -m 200,—× 400, identity ≥ 95%) was then used to map the clean data to the gene catalogs and to calculate the quantity of the genes for each sample. The gene abundance was calculated based on the total number of the mapped reads and the normalized gene length. The taxonomic assignment of the metagenomes was performed by comparing the marker gene homologs to a NCBI nonredundant NCBI-nr (ftp://ftp.ncbi.nlm.nih.gov/blast/db/) database (201,810) of taxonomically informative gene families using DIAMOND (v0.9.9.110) [33]. The homologs were annotated based on the sequence or phylogenetic similarity to the database sequences. The abundance of different taxonomic ranks was based on the gene abundance tables. As the last step, microbial functional pathways were annotated using the Kyoto Encyclopedia of Genes and Genomes (KEGG) (https://www.genome.jp/kegg/) and the evolutionary gene genealogy Non-supervised Orthologous Groups (eggNOG) database.

### Microbiome analysis

Microbiome diversity analyses were performed and visualized using phyloseq [34], vegan [35], microViz [36], and ggplot2 [37] packages in R. Species and KEGG Orthology groups (KOs) presented in > 10% of samples and with 0.01% or higher relative abundance were included in downstream analyses. Alpha-diversity was determined by the Shannon diversity index and the observed number of unique species (i.e., observed richness), using the “diversity” and “specnumber” functions from the vegan package. Case–control comparisons were tested by linear-mixed effect models (LME) to adjust for body mass index (BMI), age, frequency of antibiotics consumption in the last year, consumption of M01A (non-steroids anti-inflammatory and anti-rheumatic products), A06 (drugs for constipation), and A02BC (proton pump inhibitors) in the last 3 months, gut emptying frequency and stool consistency, with the function “aov” from the stats package [38]. Beta-diversity was represented using principal coordinate analysis (PCoA), based on the Bray Curtis dissimilarity, and tested for significance by permutational analysis of variance (PERMANOVA) using the “adonis2” function from vegan package.

To identify the differential microbial species between cases and controls, differential abundance analysis was performed using an Analysis of Compositions of Microbiomes with Bias Correction (ANCOM-BC) from the ancombc2 package [39]. ANCOM-BC models the absolute abundances using a linear regression framework [39]. Herein, absolute abundance for identified species present in > 10% of samples with > 0.01% within each phylogenetic domain (e.g., 861 bacteria, 3 archaea, 11 eukaryota, and 12 viruses) was included in the differential abundance analysis. Three taxa were unclassified at the kingdom level and removed from the analysis. Additionally, ANCOM-BC was used to examine differential KOs and eggNOG orthologs between women with endometriosis and controls.

### PAM clustering

Fecal samples were clustered by applying the Partitioning Around Medoids (PAM) algorithm, also simply referred to as k-medoids, using the “pam” function from the cluster package [40]. K-medoids consists in partitioning (clustering) the data into k clusters “around medoids,” a more robust version of K-means [41]. The number of clusters that best fits the data was selected by looking at the highest Silhouette Index, since 1 denotes the best meaning that the data point is very compact within the cluster to which it belongs and far away from the other clusters.

### Estrobolome-associated sequence reads analysis

A representative set of enzymes associated with estrogen metabolism was extracted from the atlas of human gastrointestinal microbiome-encoded enzymes from the Human Microbiome Project database [42]. This atlas identified 279 unique microbial β-glucuronidase enzymes, and revealed a functional differentiation based on the processing of distinct substrates [42]. Those enzymes that presented an accessible ID number were included in our study and their protein sequences were downloaded from the NCBI protein database [43]. Metagenomic reads were mapped to enzyme sequences using DIAMOND [33] software package with –mid-sensitive mode enabled. Alignments (reads) with < 90% query coverage were filtered out. The total number of aligned read pairs was finally reported for each enzyme involved in the analysis. To study potential alterations in these estrogen pathway-related enzymes in cases and controls, comparisons were performed using the ANOVA-Like Differential Expression tool (ALDEx2 v.1.28.1) [44].

### Statistical analyses

Descriptive characteristics of the study participants were reported as median (q1; q3) or frequency, as appropriate. BMI, age, frequency of antibiotics consumption in the last year, M01A, A06, and A02BC consumption in the last 3 months, gut emptying frequency, and stool characteristics (Bristol stool scale) were included as potential confounders in our analyses. Five women did not record data for age, 9 for antibiotics, 2 for gut emptying frequency, and 19 for stool consistency. Hence, we imputed missed data using multiple imputation method in SPSS v.28.0.1.0. For comparing non-parametric continuous data, Mann–Whitney *U* test was performed, while categorical data was analyzed by *χ* ^2^ test.

Since alterations in the gut microbiome have been widely associated with specific menopausal symptoms [21], a sensitivity analysis excluding those women with age 50 or higher was conducted to corroborate our results (*n* = 591).

All statistical analyses were performed in R (v.4.2.1) under RStudio (v.2022.07). Statistical significance was set to 0.05 for all analyses (i.e., *p*-value or *q*-value < 0.05 for analyses using Benjamini–Hochberg false discovery rate (FDR) for multiple correction).

## Results

Our study population of 1000 women consisted of a total of 136 women with endometriosis and 864 control women. The descriptive characteristics of study participants are summarized in Table 1. Study groups significantly differed for age at sample collection, M01A, A06, and A02BC consumption, being significantly higher in women with endometriosis compared to controls (FDR *p*-values < 0.05).

**Table 1:** Descriptive characteristics of the study participants

Characteristics	Endometriosis *N* = 136	Control *N* = 864	*p*-value
Age, *median [q1; q3]*	50.0 [40.8; 57.9]	45.0[36.0; 54.0]	0.005
BMI, *median [q1; q3]*	25.1 [22.2; 29.5]	24.2 [21.6; 28.6]	0.367
Frequency of antibiotics consumption, *n (%)*			
Not in the last year	79 (58.1%)	555 (64.2%)	0.887
In the last year	26 (19.1%)	139 (16.1%)	
In the last 6 months	23 (16.9%)	128 (14.8%)	
In the last month	7 (5.2%)	33 (3.8%)	
In the last week	1 (0.7%)	9 (1.0%)	
M01A consumption in the last 3 months, *n (%)*			
Yes	28 (20.6%)	74 (8.6%)	0.0003
No	108 (79.4%)	790 (91.4%)	
A06 consumption in the last 3 months, *n (%)*			
Yes	3 (2.2%)	1 (0.1%)	0.022
No	133 (97.8%)	863 (99.9%)	
A02BC consumption in the last 3 months, *n (%)*			
Yes	18 (13.2%)	48 (5.6%)	0.005
No	118 (86.8%)	816 (94.4%)	
G02BC consumption in the last 3 months, *n (%)*			
Yes	2 (1.5%)	14 (1.6%)	1
No	134 (98.5%)	850 (98.4%)	
G03A consumption in the last 3 months, *n (%)*			
Yes	7 (5.1%)	48 (5.6%)	1
No	129 (94.9%)	816 (94.4%)	
Gut emptying frequency, *n (%)*			
More than 2 times a day	21 (15.4%)	135 (15.6%)	0.940
Once a day	76 (55.9%)	495 (57.3%)	
3–6 times a week	29 (21.3%)	168 (19.4%)	
2 times a week	3 (2.2%)	12 (1.4%)	
1–2 times a week	1 (0.7%)	6 (0.7%)	
Less than once a week	0 (0.0%)	2 (0.2%)	
Irregular	6 (4.4%)	46 (5.3%)	
Stool consistency (Bristol scale), *n (%)*			
1	12 (8.8%)	63 (7.3%)	0.367
2	31 (22.8%)	138 (16.0%)	
3	22 (16.2%)	146 (16.9%)	
4	30 (22.1%)	241 (27.9%)	
5	12 (8.8%)	114 (13.2%)	
6	28 (20.6%)	147 (17.0%)	
7	1 (0.7%)	15 (1.7%)	
Parity, *n (%)*			
Yes	48 (35.3%)	369 (42.7%)	0.249
No	88 (64.7%)	495 (57.3%)	

Data presented as median [q1, q3] and frequency, as appropriate. *P*-values adjusted by Benjamini–Hochberg false discovery rate (FDR). Abbreviations: *BMI*, body mass index; *M01A*, anti-inflammatory and anti-rheumatic products, non-steroids; *A06*, drugs for constipation; *A02BC*, proton pump inhibitors; *G02B*, contraceptives for topical use; *G03A*, hormonal contraceptives for systemic use

### Microbial landscape of the study cohort

The microbiome composition and functionality of the Estonian study population were characterized by metagenomics shotgun sequencing as previously described [25, 45]. KEGG orthology (KO) refers to a classification system used to assign orthologous gene groups to organisms. Orthologs are genes in different species that evolved from a common ancestral gene. KO provides a way to organize and compare biological information across different organisms based on these orthologous groups, aiding in the understanding of functional similarities and differences in molecular pathways and biological processes [46, 47].

**Fig. 1:**
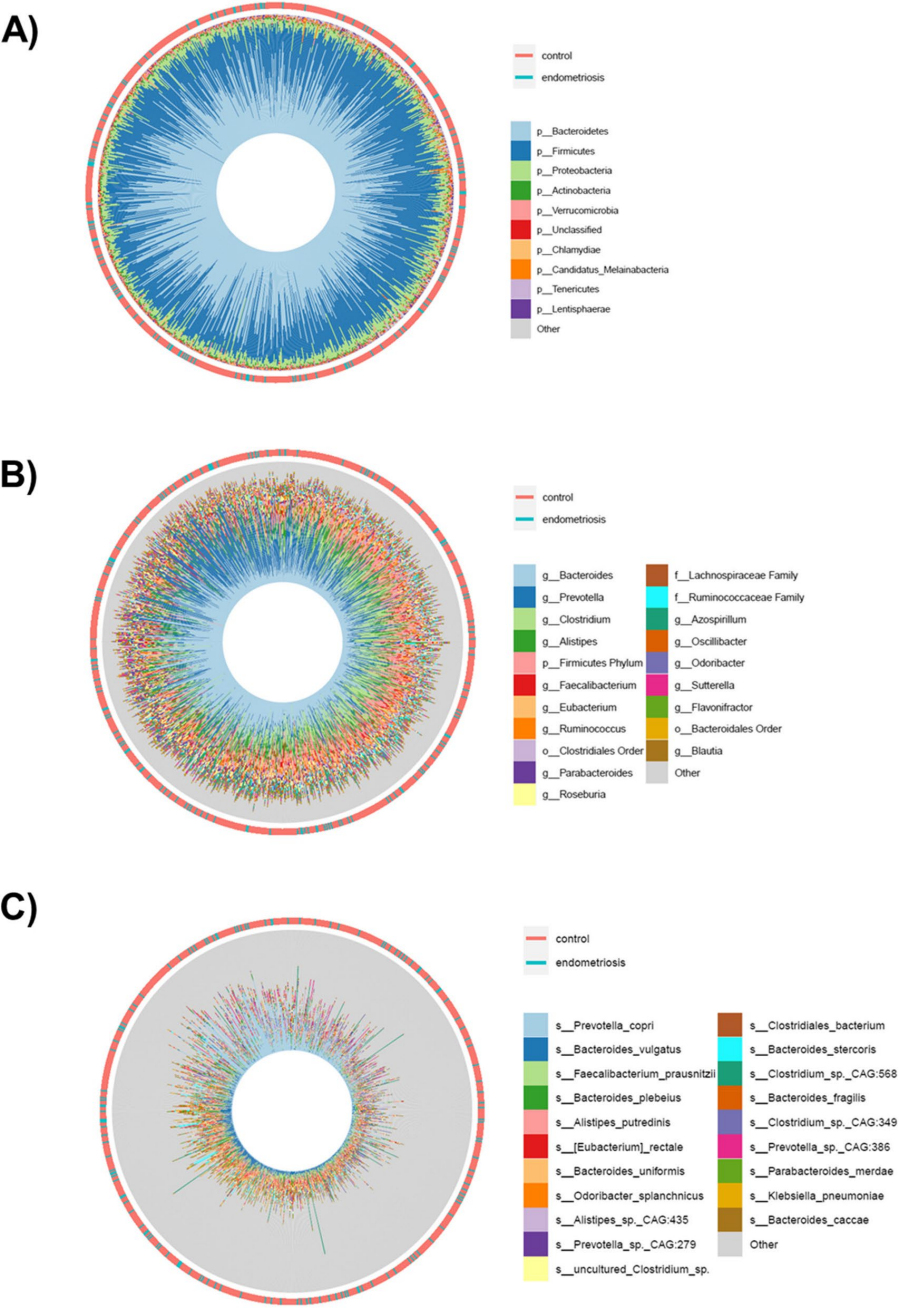
Microbial landscape in the Estonian study population. Circular stacked barplots (“iris plots”) show the most relatively abundant phyla (**A**), genera (**B**), and species (**C**) in the study population. The outer bicolor rings indicate the endometriosis and control groups

A total of 17,158 species and 7,869 KOs were detected, with an average of 6,942,273 species reads and 4,913,880 KOs reads per sample. After filtering by a prevalence > 10% and relative abundance > 0.01%, we identified 890 species and 1629 KOs. The average relative abundance of bacteria was 98.14%, followed by 0.93% for taxa of viral origin, 0.66% for eukaryotic taxa, 0.15% for archaea, and 0.13% for unclassified taxa. The most predominant phyla were *Bacteroidetes* (45.15%) and *Firmicutes* (39.86%), followed by *Proteobacteria* (7.07%), *Actinobacteria* (1.53%), and *Verrucomicrobia* (0.82%), among others (Fig. 1A). The most abundant genera consisted of *Bacteroides*, *Prevotella*, *Clostridium*, *Alistipes*, and *Faecalibacterium* (Fig. 1B). More specifically, 890 species presented > 10% prevalence and > 0.01% of relative abundance, being *Prevotella copri*, *Bacteroides vulgatus*, *Faecalibacterium prausnitzii*, *Bacteroides plebeius*, and *Alistipes putredinis* the most abundant microbes (Fig. 1C).

**Fig. 2:**
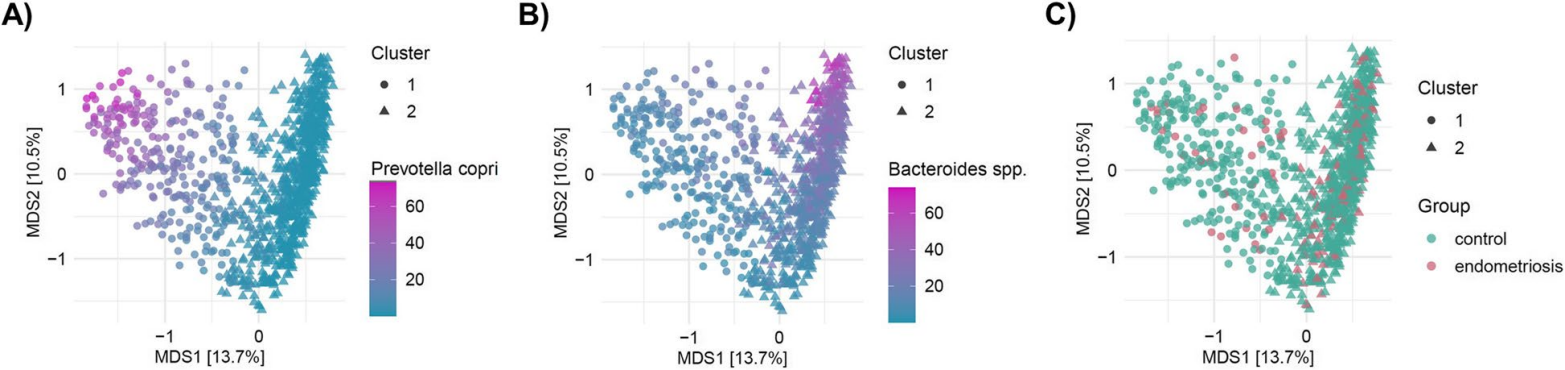
Enterotypes identified in the Estonian study population. **A**, **B** Relative abundance of *Prevotella copri* and *Bacteroides* spp. within the enterotypes on the principal coordinates analysis (PCoA) plot of the species-level microbiome profile based on the Bray–Curtis dissimilarity. **C** Distribution of women with and without endometriosis within the enterotypes. The dot’s shape indicates the cluster, while the colors highlight the relative abundances (**A**, **B**) or the endometriosis and control groups (**C**)

PAM clustering stratified the study population into two enterotypes (Additional file 1: Fig. S1), where *P. copri* and *Bacteroides* spp. drove the most significant differences in the gut microbiome (Fig. 2A, B, Additional file 1: Fig. S2). 72% of the samples were within the *Bacteroides* spp. enterotype and the remaining 28% belonged to the *P. copri* enterotype. The identified enterotypes were not correlated with the presence/absence of endometriosis, although they presented a negative correlation with BMI and positive with stool consistency (Fig. 2C; Additional file 2: Table S1).

### Microbial diversity analysis

**Fig. 3:**
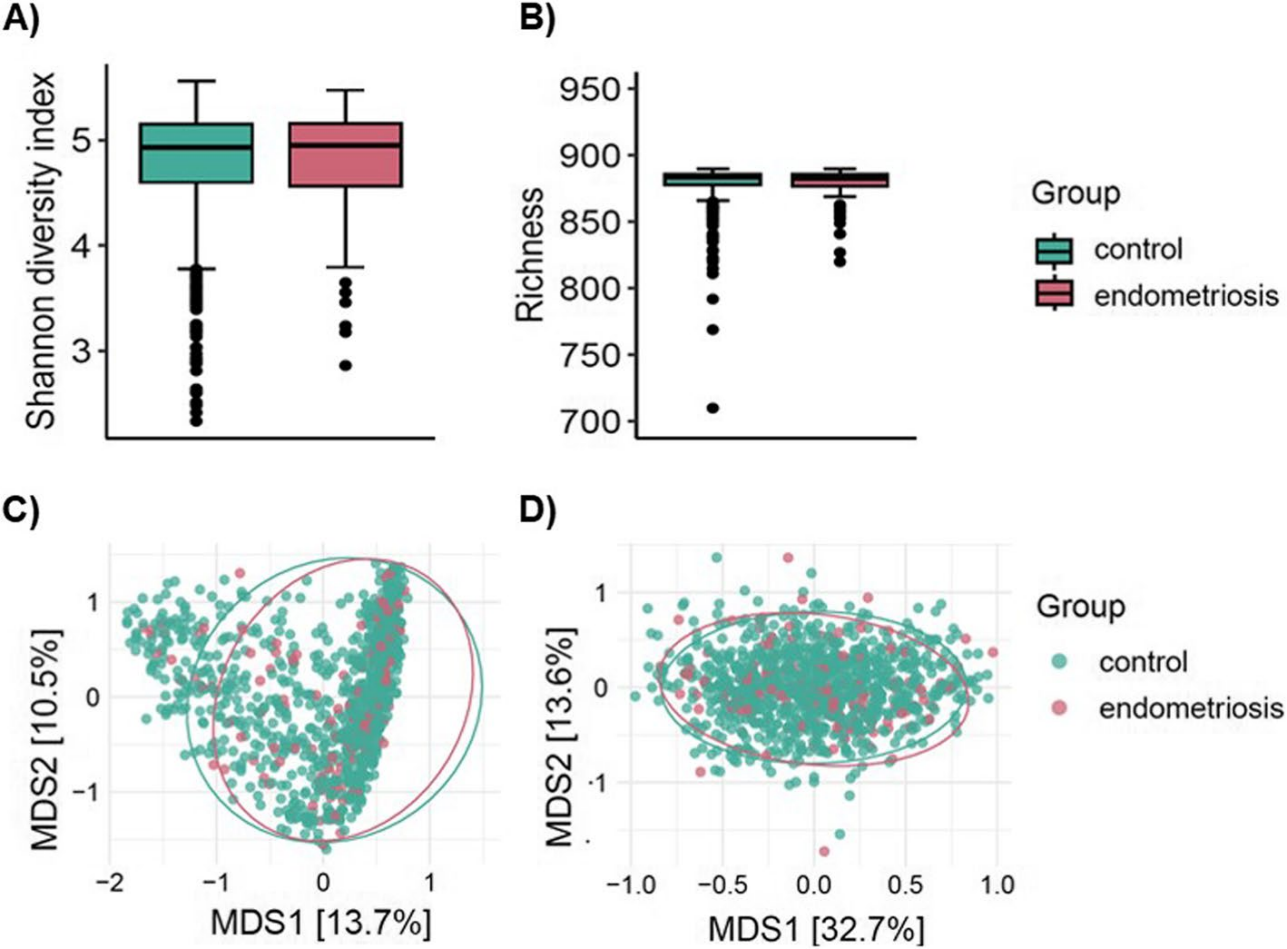
Microbial diversity measures in endometriosis and control groups. **A**, **B** Alpha-diversity analysis (i.e., Shannon diversity index and observed richness). Groups comparisons indicate no significant differences (linear-mixed effects, all *p*-values > 0.05). **C**, **D** Beta-diversity analyses on the principal coordinates analysis (PCoA) of the species (**C**) and KOs (**D**) profile based on the Bray–Curtis dissimilarity (Adonis PERMANOVA, both *R*.^2^ < 0.0007, both *p*-values > 0.05)

Next, we aimed to compare the microbial alpha- (characterized by the Shannon diversity index and observed richness) and beta-diversity between women with and without endometriosis. No significant differences between cases and controls were detected in alpha-diversity parameters, indicating that species richness was similar between both groups (all *p*-values > 0.05; Fig. 3A, B). Beta-diversity analyses on the microbial and functional profile (species and KOs profile) indicated no significant dissimilarity between the groups (PERMANOVA, both *R* ^2^ < 0.0007, *p*-values > 0.05; Fig. 3C, D). Interestingly, the strongest associations with beta-diversity both with species and KOs (all *p*-values < 0.007), were observed for the stool consistency (evaluated by the Bristol stool scale, both *R* ^2^ > 0.03), gut emptying frequency (both* R* ^2^ > 0.01), antibiotics frequency (both *R* ^2^ > 0.008), age (both *R* ^2^ = 0.004), and BMI (both *R* ^2^ > 0.003).

### Differential abundance analysis of microbial species and KOs

**Fig. 4:**
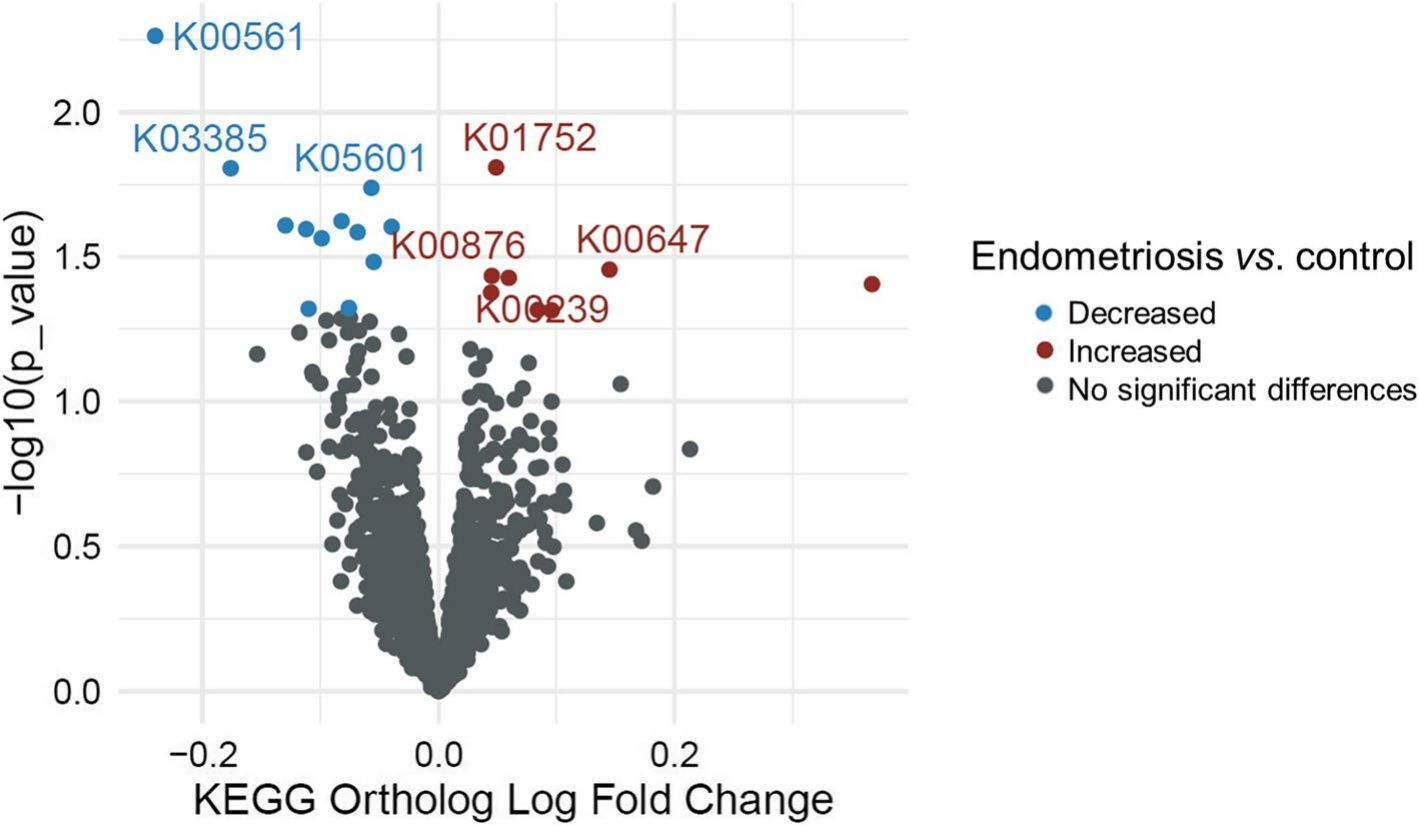
Functional differences in the microbial pathways in endometriosis and control groups. Volcano plot displaying log fold change differences in the KEGG orthologs derived from the ANCOM-BC model. Points in blue and red represent KEGG orthologs which were decreased and increased in endometriosis and statistically significant (*p* < 0.05). Points in black represent KEGG orthologs that were not significantly different (*p* > 0.05). No KEGG orthologs remained statistically significant after Benjamini–Hochberg false discovery rate (FDR) correction (all FDR *p*-values > 0.05)

To detect specific species or microbial pathways that could be potentially involved in the pathogenesis of the disease, an ANCOM-BC analysis was performed on the identified species and KOs. Overall, 28 bacteria seemed to be differentially abundant between groups, for example, *Clostridium* sp. CAG:307 (logFC = 0.595, *p* = 0.019) and *Acinetobacter* sp. CAG:196 (logFC = 0.745, *p* = 0.017) were enriched in the endometriosis group, whereas *Ruminococcus* sp. CAG:177 (logFC =  − 0.420, *p* = 0.023) and *Roseburia* sp. CAG:45 (logFC =  − 0.369, *p* = 0.005) were decreased compared to controls (Additional file 2: Table S2). Regarding functional analysis, 12 KOs associated with endometriosis, including nitrogen metabolism (logFC =  − 0.176, *p* = 0.016) or oxidative phosphorylation (logFC =  − 0.040, *p* = 0.025) that were decreased, while 8 KOs including fatty acid biosynthesis (logFC = 0.145, *p* = 0.035) and amino acids metabolism (logFC = 0.049, *p* = 0.016) were increased in women with endometriosis compared to controls (Fig. 4). However, no bacteria and KOs remained significantly different after FDR correction (all *p*-values > 0.05) (Additional file 2: Tables S2–S3). The functional analysis’ results are supported by the comparison of eggNOG orthologs, which did not reveal significant differences between the study groups (all FDR *p*-values > 0.05) (Additional file 2: Table S4).

### Sensitivity analysis

A sensitivity analysis including only women at their reproductive age (≤ 50 years) and excluding women at menopause (> 50 years) was performed to corroborate the previous results on the whole cohort. A total of 66 women with endometriosis and 525 control women were finally included. The obtained results were similar to the whole cohort results, detecting no statistically significant differences between the groups in microbial diversity and differential abundance analyses on the species and KOs profiles (Additional files 1 and 2: Fig. S3 and Tables S5–S6).

### Estrobolome pathway analysis

Since estrogen metabolism has been described as a keystone factor in the pathogenesis of proliferative disorders such as endometriosis, we analyzed key enzymes from the estrobolome that could lead to hyperestrogenic conditions. Thus, we compared the total read count of 156 estrogen pathway-related enzymes (including beta-glucuronidases and beta-galactosidases) between the women with and without endometriosis. No significant differences were detected in the total read count between the cases and controls (*p* > 0.05, Additional file 1: Fig. S4). Additionally, each enzyme was compared between groups using the ALDEx2 package (v1.28.1). We did not observe any enzyme with statistically significant differences between the endometriosis and control women (all *p*-values > 0.05, Additional file 2: Table S7). Multiple testing correction was applied for all analyses.

## Discussion

Endometriosis is a widespread gynecological disorder, and despite active research, there is a lack of understanding of the pathogenesis of the disease and its associated symptoms. Scientific evidence supports that estrogen drives the proliferation of endometrial-like lesions, although the reason why some women develop endometriosis and others do not remains unclear. Since the role of the gut microbiome in inflammatory and proliferative conditions as well as in estrogen metabolism is established [18, 21], it is rational to propose an involvement of the gut microbiome in the development of the disease. Indeed, novel studies are focusing on the gut microbial communities as important candidates for investigation in reproductive health, and several studies are associating uterine microbes with endometriosis [48–51].

To the best of our knowledge, our study is the first whole metagenome study (identifying bacteria, viruses, fungi, protozoa, and archaea) performed in women with endometriosis, while all previous studies have exclusively analyzed the 16S rRNA gene region of the bacteria. Our study results did not identify distinct compositional or functional gut microbial profiles in women with endometriosis compared to controls, which has been observed also in a previous marker gene-based study (16S rRNA gene analysis) [52]. However, other marker gene-based studies have associated several gut microbes with endometriosis [53, 54]. The largest study conducted up to date analyzed the gut microbiome profile of 66 women with endometriosis and 198 control women [53], where a higher abundance of *Parabacteroides* genus and lower *Paraprevotella* in endometriosis patients compared to controls were detected. In our study of 1000 participants, we detected a decrease in *Paraprevotella clara* and *Parabacteroides* sp. D26 in women with endometriosis, although these differences disappeared after multiple testing correction. A recent study compared the gut microbiome in 12 patients with moderate-to-severe endometriosis and 12 healthy women [54]. Although they did not describe any statistically significant differences in alpha-diversity, several genera such as *Blautia*, *Bifidobacterium*, *Dorea*, and *Streptococcus*, were significantly increased in the endometriosis group compared to controls, while *Lachnospira* and *Eubacterium eligens* group showed a decreased abundance in women with endometriosis. Another study built classification models with machine learning on the vaginal and gut microbial composition to predict rASRM stages 1–2 (minimal-to-medium) *vs*. 3–4 (moderate-to-severe) endometriosis and found that the microbe that contributes the most to this prediction was *Anaerococcus* genus [55]. In our study, species from the *Anaerococcus* genus, however, were not detected. Nonetheless, current studies are hardly comparable due to the different sample sizes and microbiome detection methods, proving contradicting and inconclusive results. Importantly, contrastingly to our study where we analyzed species level by shotgun sequencing, the previous studies performed a 16S rRNA gene analysis, which limits a reliable taxonomic assignment to genus level.

Recently, a higher frequency of *Fusobacterium* in both the endometria and ovarian endometriotic tissues from 79 patients with endometriosis was detected when compared to endometria from 76 control women [56]. Hence, they investigated further the pathogenic role of this bacteria in the development of endometriosis. Interestingly, we detected a higher relative abundance of *Fusobacterium* sp. CAG:815 in the gut in women with endometriosis, although the differences did not remain significant after adjustment for multiple comparisons.

While evidence supporting the role of the endometrial transcriptome in endometriosis development is accumulating [57, 58], a new debate is whether there are microbial pathways involved in the pathogenesis of the disease. In this context, our study identified several KOs possibly associated with the presence of endometriosis. We noted that a KO related to the long-chain saturated fatty acids biosynthesis, a metabolic pathway catalyzed by fatty acid synthase (FASN) was increased in women with endometriosis. In some cancer cell lines, based on the host gene homologs, FASN has been found to be fused with estrogen receptor, and its overexpression is a common molecular feature in hormone-sensitive cells, being regulated by both estradiol and progesterone [59]. During the menstrual cycle, FASN expression appears to be linked to endometrial cell proliferation [60, 61]. Thus, inhibiting FASN has been proposed as a therapy targeting estrogen receptor signaling in breast and endometrial cancer [62]. In fact, several studies associate the high prevalence of endometriosis with excessive lipid intake or a lipid intake imbalance and propose novel lipid metabolism-targeted approaches for the treatment of endometriosis due to the proliferative and inflammatory state of the disease [63].

We also explored the microbial genes involved in estrogen metabolism, the estrobolome, that is recognized as an important factor in the development of proliferative disorders, including endometriosis [18, 64]. Through a comprehensive analysis of 156 estrogen pathway-related enzymes, including main candidates like beta-glucuronidases and beta-galactosidases, no significant differences in any of these enzymes between the case–control groups were detected. In line with our study results, a recent study conducted enzymatic activity assays on fecal samples from women with and without endometriosis and detected no significant differences in the average level of β-glucuronidase activity between groups [64]. These findings suggest that alterations in the abundance of these specific enzymes from the estrobolome may not directly correlate with the presence of endometriosis in our studied cohort. Nevertheless, the estrogen-estrobolome-endometriosis axis is complex and our study results cannot rule out its importance in the disease development, which warrants further research.

Our study provides pioneering results about the gut microbiome composition and association with endometriosis on a large-scale study population, however, it has several limitations that should be highlighted. First, the detection power in our case–control study might have been influenced by including different subtypes of endometriosis. Endometriosis is defined as a heterogeneous disease broadly characterized into three phenotypes with different grades of severity: from superficial peritoneal as the least severe form, to ovarian and deep infiltrating endometriosis, the last being the most severe phenotype [4]. Since the inclusion of the three phenotypes could mask the presence of microbial alterations in the most severe forms, additional analyses on the different subtypes are needed to confirm our results. Furthermore, hormonal imbalance has been demonstrated to have a negative impact on the gut microbiome, while it has been reported that hormonal treatment reverses the gut microbiome dysbiosis in reproductive disorders [65]. Since the use of estrogen-progestins and progestins is the first-line medical treatment in endometriosis [19], patients with hormonal treatment may present similar gut microbial profiles than those without the disease. Hence, more studies on women with active endometriosis and no hormonal treatment are warranted to unravel the complex bidirectional relationship between the gut microbiome and endometriosis.

## Conclusions

The molecular mechanisms underlying the pathogenesis of endometriosis are not yet fully understood, which presents a challenge in its diagnosis and treatment. In this context, the gut microbiome emerges as a potential diagnostic tool and therapeutic target. We present the largest whole metagenome study on endometriosis so far; however, our study findings do not provide enough evidence to support the existence of a gut microbiome-dependent mechanism implicated in the pathogenesis of endometriosis. Further research, especially involving large-scale study populations with active endometriosis and without hormonal treatment, is crucial to better understand the endometriosis-associated microbiome, the microbiome-immune response axis, and to unravel its potential for diagnosis and treatment approaches.

## Supplementary Information

Additional file 1: Figures S1-S4. FigS1- Enterotypes model fit by the number of clusters. 2 clusters were selected as an optimal number based on the highest Silhouette Index. FigS2- Heatmap illustrating the top 20 most abundant species of the Estonian study population. The taxa are rank-ordered with the most abundant taxon on the left in the x-axis. Participants are displayed in the y-axis. FigS3- Sensitivity analysis of microbial diversity measures in endometriosis and control groups. (A, B) Alpha-diversity analysis (i.e., Shannon diversity index and observed richness) after excluding women with age > 50. Groups comparisons indicate no significant differences (Linear-mixed effects, all *p*-values > 0.05). (C, D) Beta-diversity analyses on the principal coordinates analysis (PCoA) of the species (C) and KOs (D) profile based on the Bray–Curtis dissimilarity (Adonis PERMANOVA, both R2 > 0.001, both *p*-values > 0.05). FigS4- Estrobolome analysis comparing endometriosis and control groups. Total read count from estrogen-related enzymes was not significantly different between groups (Mann Whitney U test: *p*-value > 0.05).

Additional file 2: Tables S1-S7. Table S1- Correlation analysis between gut enterotypes and clinical factors. Table S2- Differential abundance analysis in endometriosis and control groups. Species with a prevalence > 10% and relative abundance ≥ 0.1% were compared in endometriosis and control groups using an Analysis of Compositions of Microbiomes with Bias Correction (ANCOM-BC). Table S3- Differential abundance analysis in endometriosis and control groups. KEGG orthologs (KO) with a prevalence > 10% and relative abundance ≥ 0.1% were compared in endometriosis and control groups using an Analysis of Compositions of Microbiomes with Bias Correction (ANCOM-BC). Table S4- Differential abundance analysis in endometriosis and control groups. EggNOG orthologs with a prevalence > 10% and relative abundance ≥ 0.1% were compared in endometriosis and control groups using an Analysis of Compositions of Microbiomes with Bias Correction (ANCOM-BC). Table S5- Sensitivity differential abundance analysis in endometriosis and control groups. Species with a prevalence > 10% and relative abundance ≥ 0.1% were compared in endometriosis and control groups after excluding women with age > 50, using an Analysis of Compositions of Microbiomes with Bias Correction (ANCOM-BC). Table S6- Sensitivity differential abundance analysis in endometriosis and control groups. KEGG orthologs (KO) with a prevalence > 10% and relative abundance ≥ 0.1% were compared in endometriosis and control groups after excluding women with age > 50, using an Analysis of Compositions of Microbiomes with Bias Correction (ANCOM-BC). Table S7- Estrogen path enzymes with ALDEx2 analysis between endometriosis and control groups.

## Data Availability

The metagenomic data analyzed during the current study are available in the European Genome-Phenome Archive database (https://www.ebi.ac.uk/ega/) under accession code EGAS00001008448.

## Abbreviations

ABC: ATP-binding cassette
ANCOM-BC: Analysis of Compositions of Microbiomes with Bias Correction
BMI: Body mass index
FASN: Fatty acid synthase
KEGG: Kyoto Encyclopedia of Genes and Genomes
KO: KEGG Orthology group
LME: Linear-mixed effect models
PAM: Partitioning Around Medoids
PCoA: Principal coordinates analysis
PERMANOVA: Permutational analysis of variance
