# Correction: Gut microbiome in endometriosis: a cohort study on 1000 individuals

**DOI:** 10.1186/s12916-024-03692-6

**Published:** 2024-10-10

**Authors:** Inmaculada Pérez-Prieto, Eva Vargas, Eduardo Salas-Espejo, Kreete Lüll, Analuce Canha-Gouveia, Laura Antequera Pérez, Juan Fontes, Andres Salumets, Reidar Andreson, Oliver Aasmets, Estonian Biobank research team, Katrine Whiteson, Elin Org, Signe Altmäe

**Affiliations:** 1 Department of Biochemistry and Molecular Biology I, Faculty of Sciences, University of Granada, Granada, Spain; 2 Instituto de Investigación Biosanitaria Ibs.GRANADA, Granada, Spain; 3 Systems Biology Unit, Department of Experimental Biology, Faculty of Experimental Sciences, University of Jaen, Jaen, Spain; 4 Institute of Genomics, Estonian Genome Centre, University of Tartu, Tartu, Estonia; 5 Department of Physiology, Faculty of Veterinary, University of Murcia, Campus Mare Nostrum, Murcia, Spain; 6 Department of Computer Engineering, Automation and Robotics, University of Granada, Granada, Spain; 7 U. Reproducción, UGC Laboratorio Clínico y UGC Obstetricia y Ginecología. HU Virgen de Las Nieves, Granada, Spain; 8 Competence Centre On Health Technologies, Tartu, Estonia; 9 Division of Obstetrics and Gynaecology, Department of Clinical Science, Intervention and Technology, Karolinska Institutet, Huddinge, Stockholm, Sweden; 10 Department of Gynaecology and Reproductive Medicine, Karolinska University Hospital, Huddinge, Stockholm, Sweden; 11 Department of Obstetrics and Gynaecology, Institute of Clinical Medicine, University of Tartu, Tartu, Estonia; 12 Institute of Molecular and Cell Biology, University of Tartu, Tartu, Estonia; 13 School of Biological Sciences, University of California, Irvine, CA USA

**Correction** **:** **BMC Med 22, 294 (2024)**

**https://doi.org/10.1186/s12916-024-03503-y**

The original article [1] presents incorrect institutions for affiliations #5, #9, #10, #11 and #12. The corrected affiliations can be viewed in this Correction article.

Corrected affiliations:

#5 Department of Physiology, Faculty of Veterinary, University of Murcia, Campus Mare Nostrum, Murcia, Spain.

#9 Division of Obstetrics and Gynaecology, Department of Clinical Science, Intervention and Technology, Karolinska Institutet, Huddinge, Stockholm, Sweden.

#10 Department of Gynaecology and Reproductive Medicine, Karolinska University Hospital, Huddinge, Stockholm, Sweden.

#11 Department of Obstetrics and Gynaecology, Institute of Clinical Medicine, University of Tartu, Tartu, Estonia.

#12 Institute of Molecular and Cell Biology, University of Tartu, Tartu, Estonia.
