# Supplementary material for: Gut microbiome in endometriosis: a cohort study on 1000 individuals
Source: BMC Med. 2024 Jul 18;22:294. doi: 10.1186/s12916-024-03503-y (PMC11256574; doi:10.1186/s12916-024-03503-y)
Supplement: Supplementary file 1 — Additional file 1: Figures S1-S4. FigS1- Enterotypes model fit by the number of clusters. 2 clusters were selected as an optimal number based on the highest Silhouette Index. FigS2- Heatmap illustrating the top 20 most abundant species of the Estonian study population. The taxa are rank-ordered with the most abundant taxon on the left in the x-axis. Participants are displayed in the y-axis. FigS3- Sensitivity analysis of microbial diversity measures in endometriosis and control groups. (A, B) Alpha-diversity analysis (i.e., Shannon diversity index and observed richness) after excluding women with age > 50. Groups comparisons indicate no significant differences (Linear-mixed effects, all p-values > 0.05). (C, D) Beta-diversity analyses on the principal coordinates analysis (PCoA) of the species (C) and KOs (D) profile based on the Bray–Curtis dissimilarity (Adonis PERMANOVA, both R2 > 0.001, both p-values > 0.05). FigS4- Estrobolome analysis comparing endometriosis and control groups. Total read count from estrogen-related enzymes was not significantly different between groups (Mann Whitney U test: p-value > 0.05). [file 12916_2024_3503_MOESM1_ESM.docx]

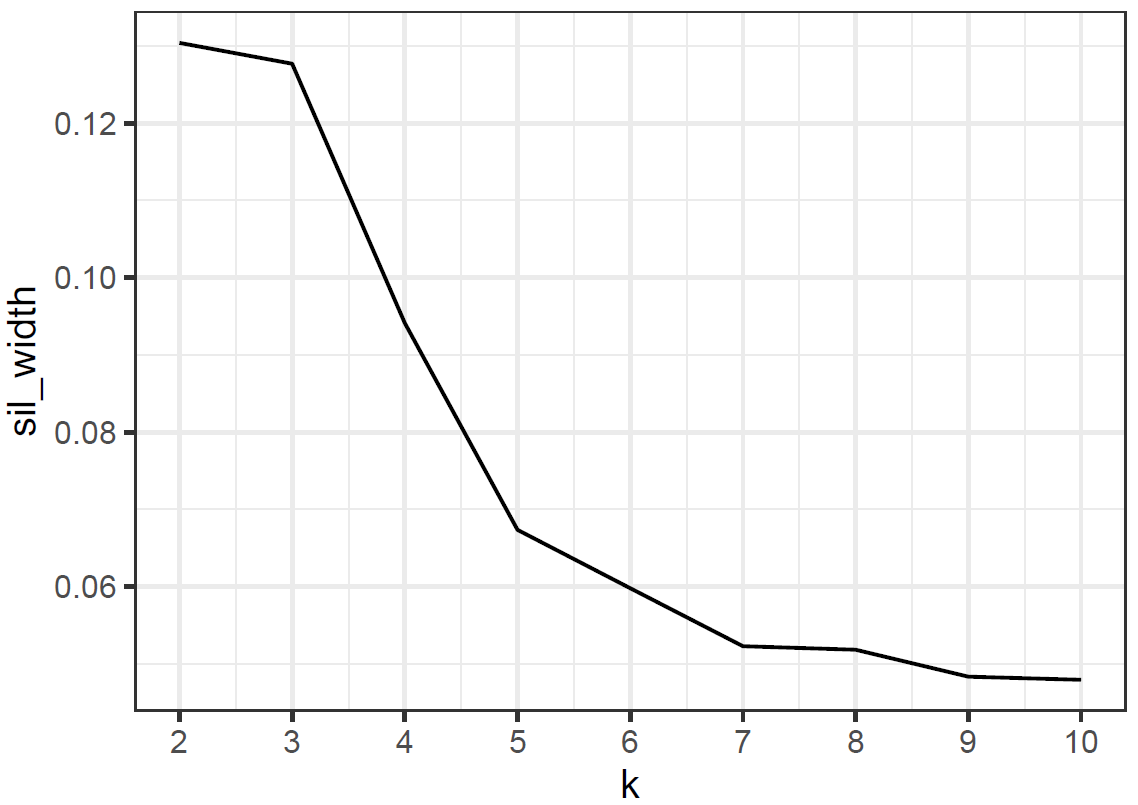
**Figure S1. Enterotypes model fit by the number of clusters.** 2 clusters were selected as an optimal number based on the highest Silhouette Index. Abbreviations: sil_width: Silhouette width; k: clusters.


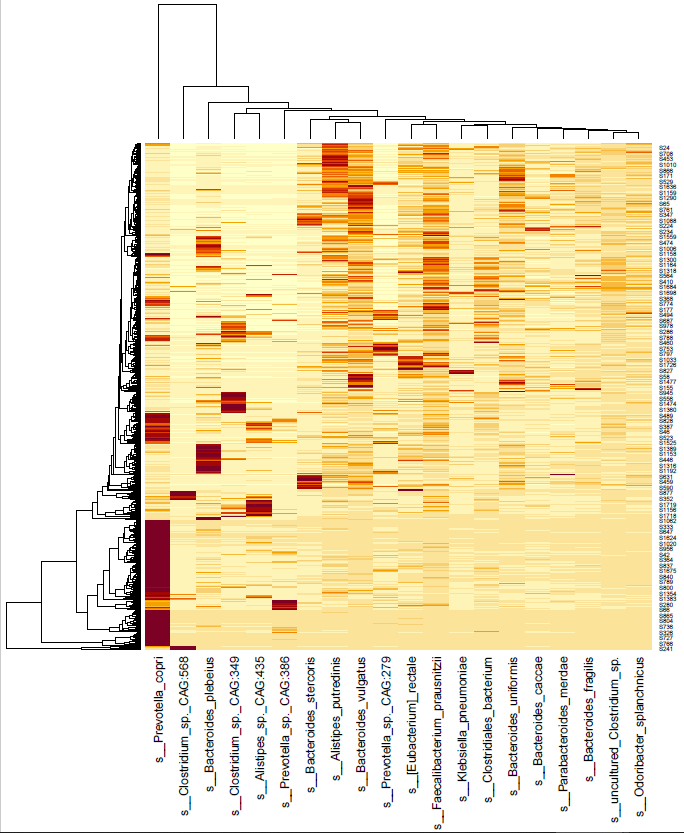


**Figure S2. Heatmap illustrating the top 20 most abundant species of the Estonian study population.** The taxa are rank-ordered with the most abundant taxon on the left in the x-axis. Participants are displayed in the y-axis.


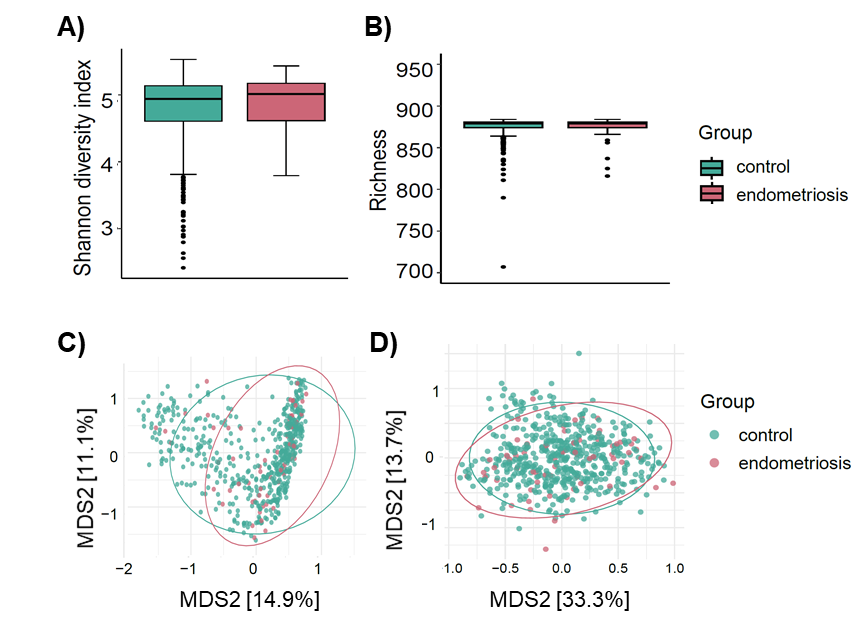
**Figure S3. Sensitivity analysis of microbial diversity measures in endometriosis and control groups.** (A, B) Alpha-diversity analysis (i.e., Shannon diversity index and observed richness) after excluding women with age >50. Groups comparisons indicate no significant differences (Linear-mixed effects: all p-values >0.05). (C, D) Beta-diversity analyses on the principal coordinates analysis (PCoA) of the species (C) and KOs (D) profile based on the Bray-Curtis dissimilarity (Adonis PERMANOVA, both R^2^ >0.001, both p-values >0.05).

**
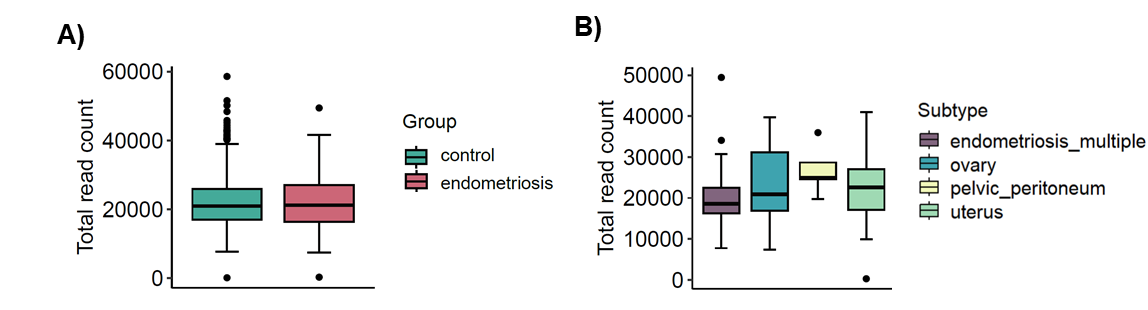
Figure S4. Estrobolome analysis comparing endometriosis and control groups**. Total read count from estrogen-related enzymes was not significantly different between groups (Mann Whitney *U* test: p-value >0.05).
